# A novel T-cell proliferation-related model for predicting the prognosis of head and neck squamous cell carcinoma

**DOI:** 10.1097/MD.0000000000041657

**Published:** 2025-02-28

**Authors:** Wenkai Huang, Mingyu Zhao, Yunshan Li, Junwei Xiang, Lin Yang, Yuanyin Wang, Ran Chen

**Affiliations:** a College & Hospital of Stomatology, Anhui Medical University, Key Laboratory of Oral Diseases Research of Anhui Province, Hefei City, China.

**Keywords:** HNSCC, immune, prognostic model, T-cell proliferation biomarker, TCGA, tumor subtype

## Abstract

Head and neck squamous cell carcinoma (HNSCC) have a poor prognosis since its high rates of metastasis and recurrence. T-cell proliferation-related genes (TRGs) act a significant role in tumor pathology through regulating the function, proliferation of immune cells. We designed and validated an individualized TRGs signature for predicting prognosis in HNSCC patients with risk estimation model. We screened out differentially expressed TRGs (DETRGs) in cancer tissues as opposed to paracancerous tissue. gene ontology and Kyoto Encyclopedia of Genes and Genomes pathway analyses were used to investigate the functional involvement of TRGs in the TGCA HNSCC cohort. We constructed a TRG signature using 7 biomarkers which screened by univariate and multivariate analysis and reclassified the HNSCC patients into high- and low-risk group according to prognostic information. After Kaplan–Meier analyzing, we found that patients in high risk was extremely lower in survival than patients in low risk. Combining univariate and multivariate regression analysis, we prove that risk scores is an independent prognostic factor. Further, we explored the immune function and tumor mutation burden (TMB) of our prognostic model. Functional enrichment analyses suggested that TRGs mainly included in the biological pathways related to T-cell and other immune cell response. Different tumor microenvironment, immune cells and TMB can be distinguished clearly according to both risk stratification and subtype clustering. In this study, our team successfully identified specific T-cell proliferation-related genetic biomarkers of HNSCC and established a new prognostic model of HNSCC based on TRGs, which has the outstanding performance in predicting the prognosis of HNSCC.

## 1. Introduction

The most prevalent head and neck malignancy is head and neck squamous cell carcinoma (HNSCC), which comes from the mucosal epithelium of the oral cavity, throat, and larynx.^[1]^ Meanwhile, HNSCC is the sixth most prevalent cancer globally, with approximately 500,880,000 patients diagnosed with the disease each year.^[2]^ Smoking, alcohol, and human papillomavirus infection are the most common predisposing factors. In addition, betel nut chewing, poor oral health and chronic hepatitis C infection also promote the incidence of HNSCC.^[3]^ HNSCC tissues are immunosuppressed, including particular antigens, less lymphocytes and NK cells than normal tissues, and can avoid immune monitoring via various mechanisms.^[4]^ HNSCC is treated using a variety of modalities, including surgery, radiotherapy, and chemotherapy.^[5]^ However, the asymptomatic nature of HNSCC and the lack of early detection lead to the 5-year survival rate <50%, and approximately 30% to 40% of patients may experience failure cases of local recurrence and distant metastases after treatment.^[6]^ Suboptimal preclinical models and a lack of precise biomarkers for early cancer diagnosis have hampered the effective clinical care of HNSCC.

Previous literatures have found that cancer cells may elude antitumor immunity with regulating T-cell stimulation.^[7]^ Cancer immunotherapy could benefit tumor patients utilizing targeting tumor-specific T-cells, nevertheless the clinical effectiveness of cancer immunotherapy is remarkable in various tumor types. The variation of status and richness of T-cells depend upon the tumor microenvironment (TME) of various tumor types, which could influence clinical effectiveness, like drug reactions to immunotherapies.^[8]^ As a result, it is essential to look at the prognostic influence of T-cell proliferation-related genes (TRGs) for the purpose of overcoming tumor immune escape and find novel and useful prognostic biomarkers for HNSCC.

In this study, we required the data of RNA-Seq and clinical information of HNSCC from The Cancer Genome Atlas (TCGA). We analyzed the expression of DETRGs in HNSCC samples to identify the pathways of enrichment and their biological functions. We further screened for genes closely associated with survival in HNSCC and established a risk model in view of 7 TRGs. In addition, risk score was an independent indicator for predicting prognosis of HNSCC suffers, when we combined with clinical characteristics. We also researched the relation among the risk model and tumor-infiltrating immune cells (TICs) and immune function. Our research demonstrated that TRGs have a significant impact on HNSCC progression and are potential prognostic markers and therapeutic targets for HNSCC.

## 2. Methods

### 2.1. Requirement and processing of data

497 HNSCC samples and 44 adjacent normal samples’ RNA-seq data were downloaded from TCGA (https://tcga-data.nci.nih.gov/tcga/). And “limma” package was used for selecting genes which expressed differentially (DEGs) among normal and tumor samples based on their genetic properties. *P*-value < .05 as well as |log2fold change| >1 are our selection criteria. We extracted 207 TRGs from the previous literature and display them in Table S1, Supplemental Digital Content, http://links.lww.com/MD/O431. The data were then intersected with the above DEGs to obtain the DETRGs.

### 2.2. Protein–protein interaction network construction

The STRING database (http://string-db.org/) was used to analyze the interactions of different DETRGs and visualize interaction networks.

### 2.3. Gene ontology and Kyoto gene and genomic enrichment analysis

DETRGs are analyzed through gene ontology (GO) and Kyoto gene and genomic (KEGG) for analyzing functional enrichments, including molecular functions, cellular components, and biological pathway.

### 2.4. Construction and validation of prognostic models

Univariate Cox regression analysis was examined for the association between prognosis in patients with HNSCC and genes. Each DETRG with *P*-value < .05 was finally chosen as a candidate biomarker for further analysis. To avoid overfitting, Least Absolute Shrinkage and Selection Operator (LASSO) regression analysis was used, and the most suitable prognostic DETRG was identified. Then, an optimized risk score was constructed by a multivariate Cox regression analysis. The risk score of patients with HNSCC was estimated as follows: risk score = (where Xi is the risk factor and Yi is the expression level of each gene). We plotted receiver operating characteristic curves for the model at 1, 2, and 3 years. Chi-square test was used to verify the feasibility of the model for the clinical application and to investigate the association between clinicopathological features and risk scores. Band plots were drawn for visualization (*P* < .05 = *, *P* < .01 = **, and *P* < .001 = ***). We utilized the Kaplan–Meier plotter (https://www.kmplot.com/analysis/index.php?p=service&cancer=pancancer_rnaseqhttps://www.kmplot.com/analysis/index.php?p=service&cancer=pancancer_rnaseq) to draw the K–M plot. The tumor type was selected as HNSCC and the genes symbol were selected as 7 TRGs. The Wilcoxon signed-rank test was further used to reveal differences in risk scores between groups by clinical characteristics, and the findings are presented in box plots. To demonstrate the reliability of risk score as an independent prognostic indicator, we processed univariate Cox regression analyses as well as multivariate Cox regression analyses among RiskScore and other clinical factors. The results were presented using forest maps. R packages “pHeatmap,” “ggupbr” as well as “Survival” were used in these operations.

### 2.5. Assessment of immune microenvironment

We used well-established techniques, including TIMER, EPIC, QUANTISEQ, XCELL, CIBERSORT, CIBERSORT-ABS, and MCPCOUNTER, to assess TICs in samples to investigate whether a relation between risk scores and immune cells in HNSCC patients. With Spearman correlation analysis, we estimated the relationship among TICs and risk scores, and the relative coefficient is visualized by lollipop plot. The R package “ggplot 2” was used in this operation. Then, the expression of immune check-point genes in different groups was assessed using R “limma,” “reshape2,” “ggplot2,” and “ggpubr” packages.

### 2.6. Consensus clustering analysis

We classified all case of TCGA cohort into 2 groups in view of the consensus prognostic TRGs’ expression in tumors using the R software “ConsensusClusterPlus.” Then, using the R package “survival” to plot survival curves and “scatterplot3d” R packages to processed 3D principal component analysis (PCA).

### 2.7. Chemotherapeutic drug sensitivity analysis

To assess the value of feature biomarkers in predicting treatment effect in HNSCC, we calculated several common chemotherapeutics and molecularly targeted drugs (Cisplatin, 5-Fluorouracil, Cyclophosphamide, Carmustine, Dactinomycin, Savolitinib)’ sensitivity per sample applying pRRophetic. The Wilcoxon signed-rank test compared sensitivity differences between various groups, and the results are shown as box plots using the “ggplot2” R package.

### 2.8. Somatic mutation analysis and tumor mutation burden

The somatic mutations of TRGs involved in constructing the prognostic model were obtained from the cBioPortal database (https://www.cbioportal.org/). According to the“VarScan2 Variant Aggregation and Masking” data downloaded through UCSC Xena, the differentially expressed TRGs mutations of patients in high- and low-risk groups were analyzed and visualized by using “GenVisR” R package.Representing the number of mutations per million bases in tumor tissue, We exhibited the mutation of HNSC suffers in the TCGA cohort and computed the score of tumor mutation burden (TMB) for each patient by using “maftools,” “TCGAbiolinks,” “AnnotationDbi,” “tidyverse,” “SummarizedExperiment,” and “org.Hs.e.g..db”R packages.

### 2.9. Statistical analysis

Each experiment was repeated thrice, independently. Data are presented as the mean ± SD (standard deviation). For data analysis, the 2-tailed Student *t*-test was applied. *P* < .05 was deemed a statistically significant difference.

## 3. Results

### 3.1. Identification of DETRGs among tumor and normal groups

**Figure 1.**
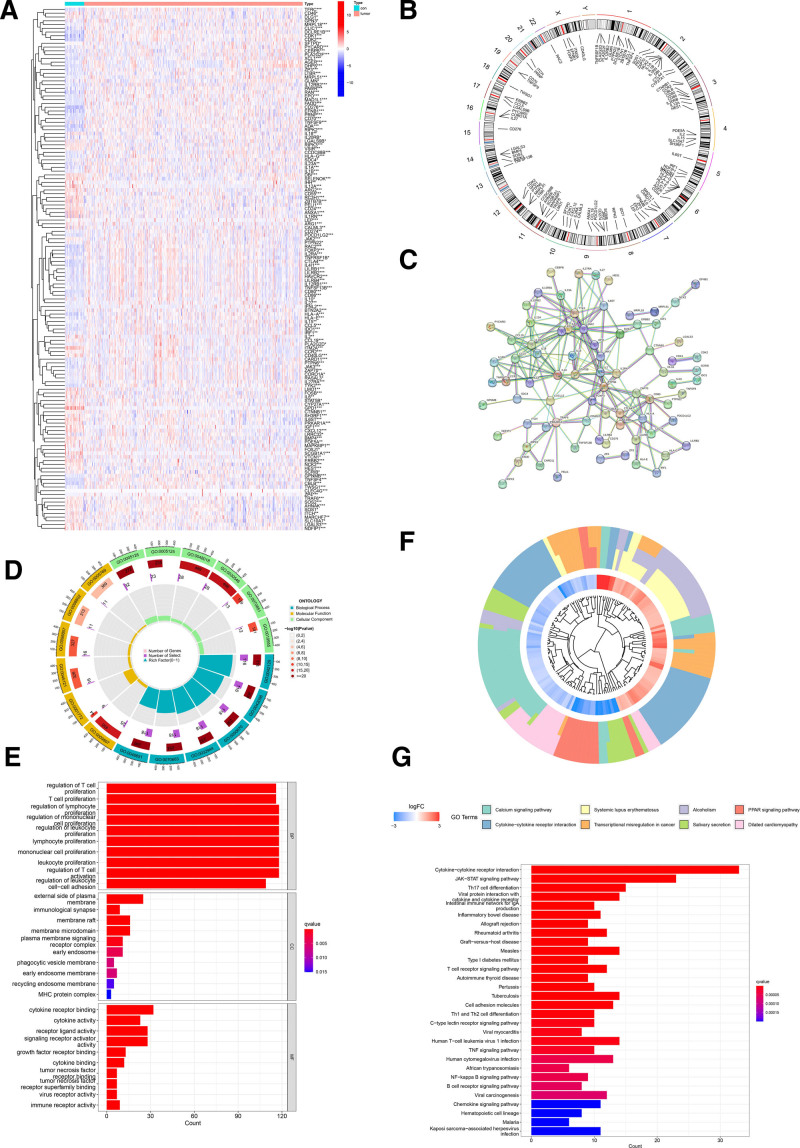
Identification and pathway analysis of T cell proliferation-related biomarkers from the TCGA cohort. (A) The heatmap of 139DETRGs. (B) CNV position on chromosome of DETRGs in TCGA cohort. (C) The PPI network indicated the interactions between these biomarkers. (D and E) The GO enrichment analysis of DETRGs. (F and G) The KEGG pathway enrichment analysis of DETRGs. CNV = copy number variant, DETRGs = differentially expressed T-cell proliferation-related genes, GO = gene ontology, KEGG = Kyoto gene and genomic, PPI = protein–protein interaction, TCGA = the cancer genome atlas.

The expression of the 139 DETRGs in normal and tumor tissues are shown in the heatmap plot (Fig. 1A). Our team identified 139 alterations in DETRGs correlated with CNVs on chromosomes (Fig. 1B). To explore the interactions between each DETRG, all DETRGs were submitted to the STRING database, which is known for its protein–protein interaction. After removing the isolated DETRGs and setting the highest confidence, the protein–protein interaction network of DETRGs is displayed, including 139 nodes and 191 edges (Fig. 1C).

### 3.2. Gene ontology function annotation and KEGG pathway analysis

We analyzed 139 DETRGs for GO and KEGG analysis and visualized the data in R4.2.1. The following functions are significantly enriched based on these DETRGs: regulation of T-cell proliferation, regulation of T-cell activation, T-cell proliferation, regulation of lymphocyte proliferation, regulation of mononuclear cell proliferation (Fig. 1D and E). KEGG pathway analysis results show that it is mainly enriched in the Th17 cell differentiation, JAK − STAT signaling pathway, Cytokine − cytokine receptor interaction (Fig. 1F and G).

### 3.3. Construction of T-cell proliferation-associated prognostic model

**Figure 2.**
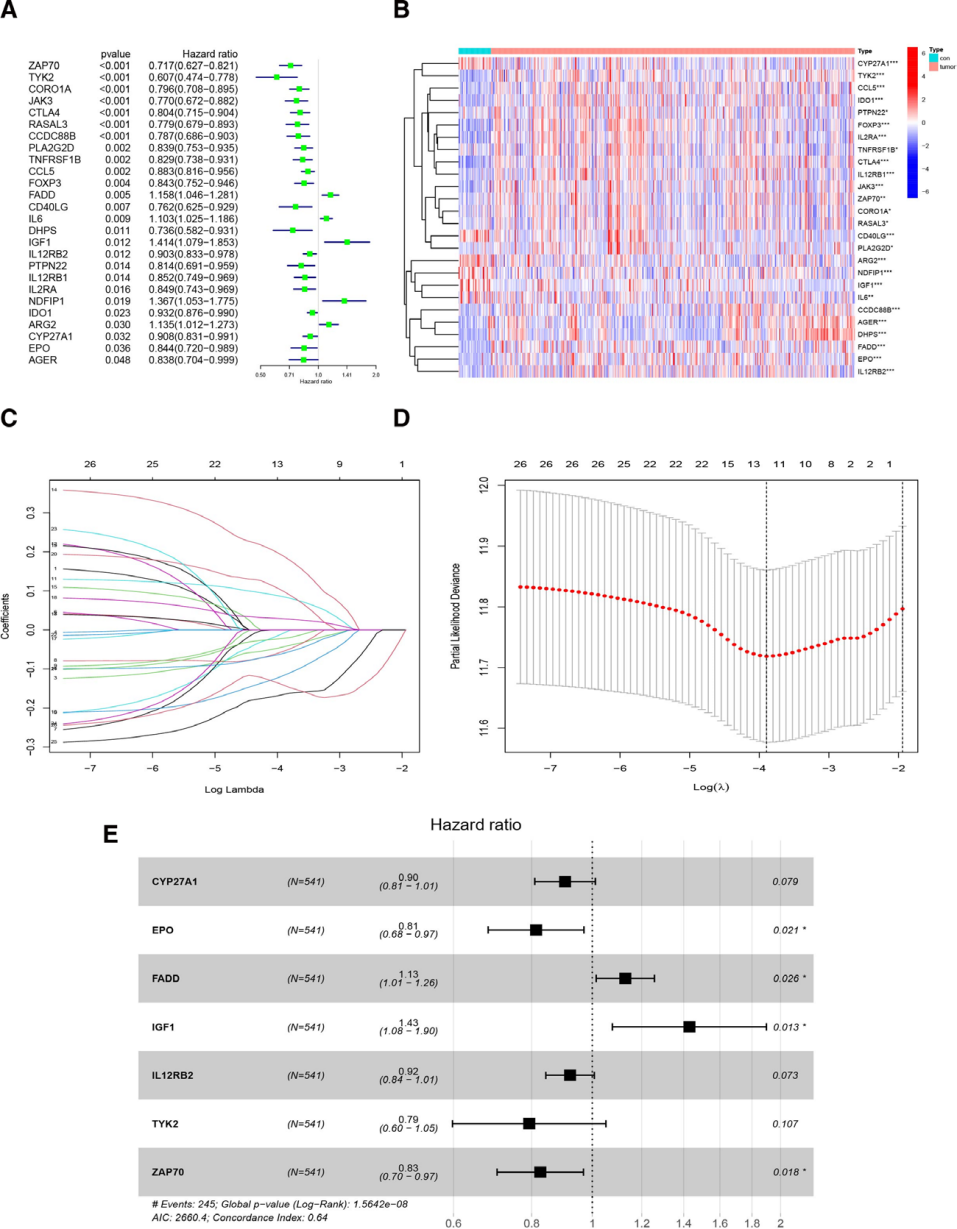
The establishment of the model. (A) 26 prognostic TRGs screened from 139 differentially expressed TRGs utilizing univariate Cox regression analysis; (B) heatmap plot of 26 prognostic TRGs; (C) the LASSO coefficient of 26 prognostic TRGs; (D) variable selection by the 10-fold cross-validation in the LASSO regression model. (E) forest plot of 7 prognostic TRGs screened from 12 LASSO TRGs with multivariate Cox regression analysis. TRGs = T-cell proliferation-related genes.

Applying univariate Cox regression (*P* < .05), we screened out 26 TRGs from 139 DETRGs (Fig. 2A and B). After that, 12 genes were kept from the LASSO regression analysis based on the 26 TRGs (Fig. 2C and D). Finally, we use multivariate Cox regression analysis which is a stepwise regression approach to screen 7 TRGs for developing an HNSCC prognostic model (Fig. 2E).

**Figure 3.**
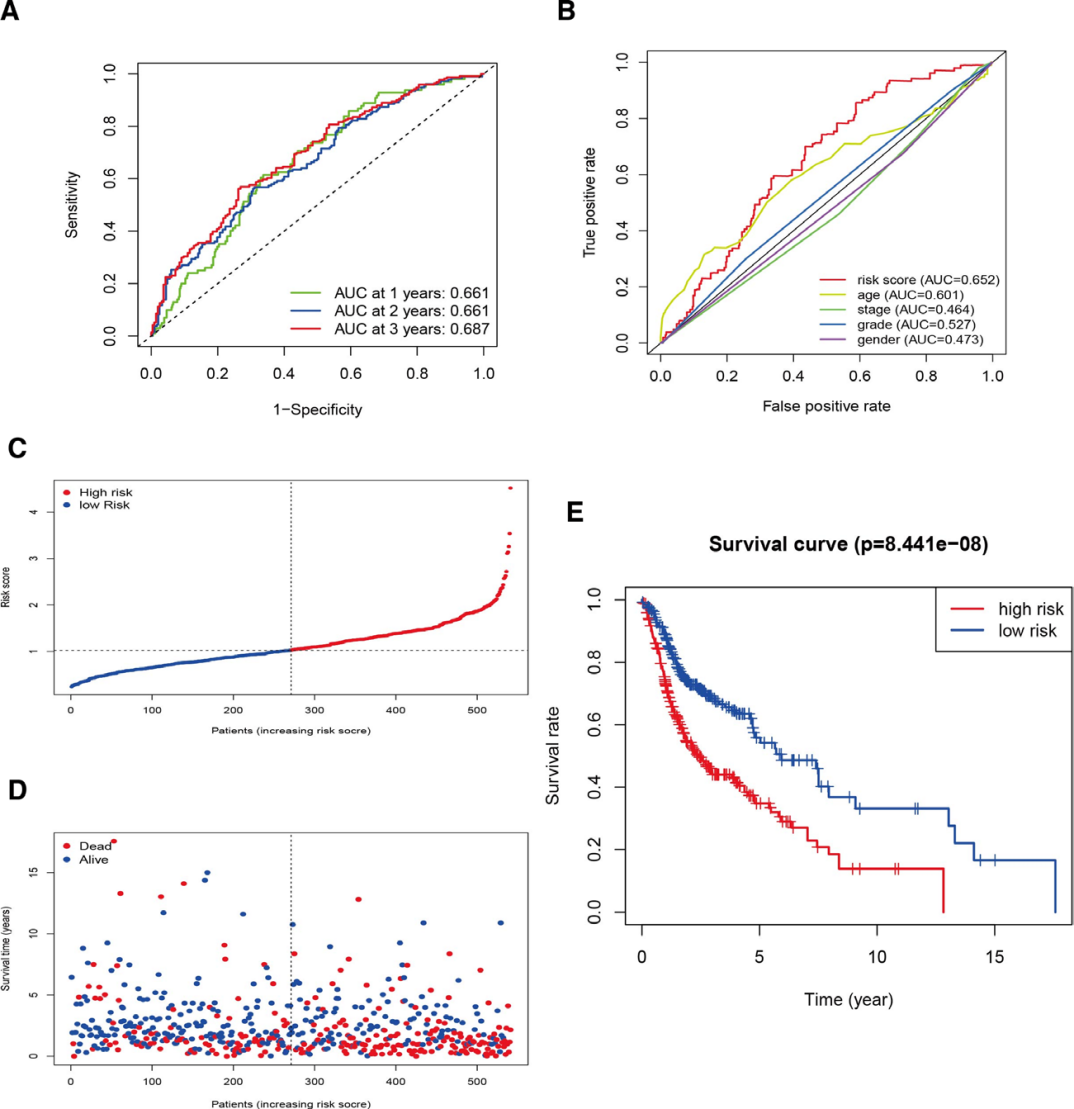
Estimation of the risk model for predicting prognosis. (A) The ROC curves of the 1-, 2-, and 3-year. (B) The 1-year ROC curve of age, stage, grade, risk score and gender. (C and D) The landscape of risk score, status and survival time. (E) The OS curves of the low-risk groups and high-risk groups are significant differences (*P* < .001). OS = overall survival, ROC = receiver operating characteristic.

We performed 1-year, 2-year, and 3-year receiver operating characteristic curves to verify the accuracy of model, and the values of AUC were all more than 0.65 (Fig. 3A). Relative to other clinicopathological features, the current risk model had the most significant predictive power, due to a max AUC value of 0.652 (Fig. 3B).

### 3.4. The model of clinical assessment through risk evaluation

**Figure 4.**
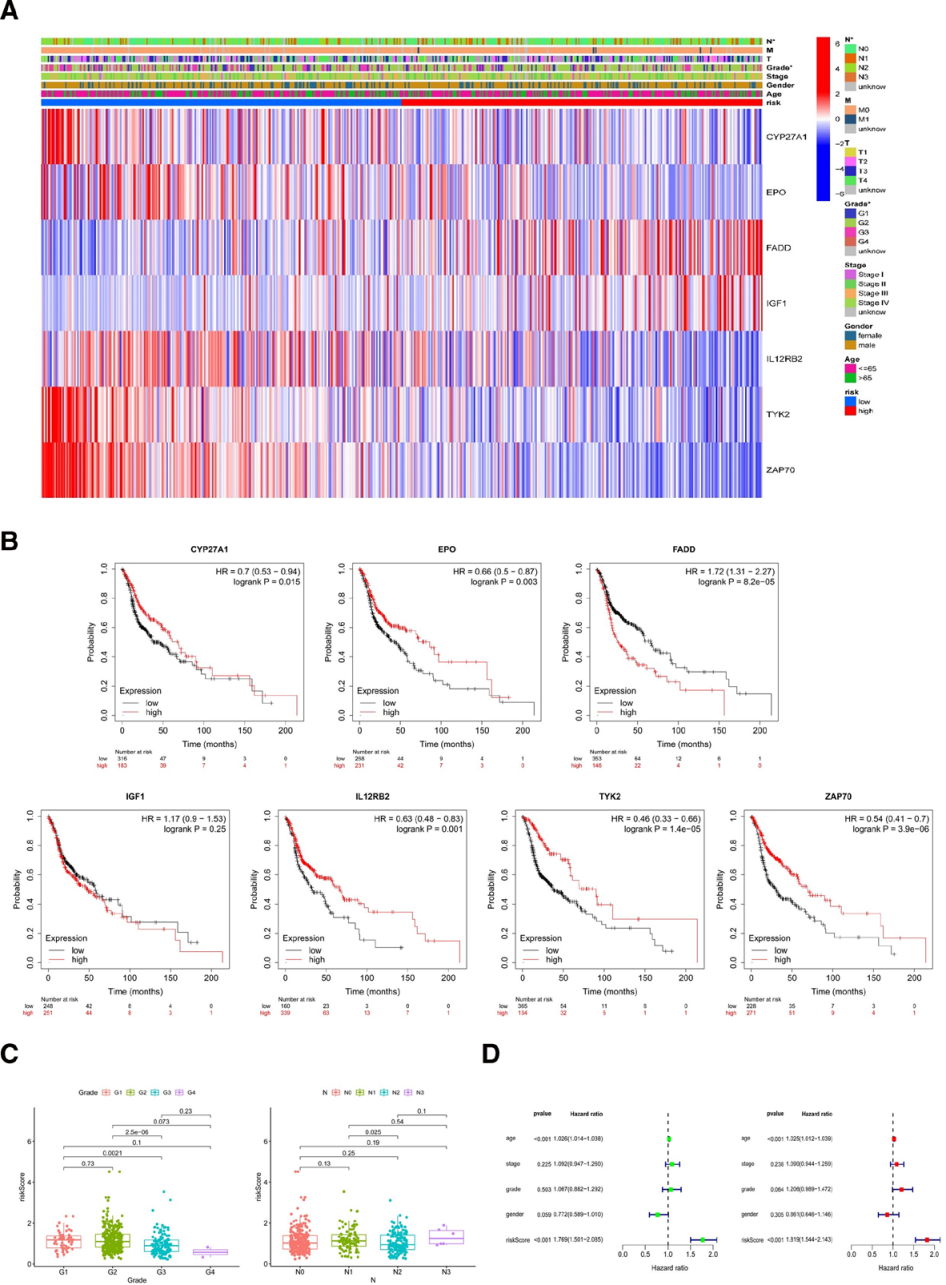
Clinical evaluation of prognostic model. (A) The clinicopathologic characters and heatmap classified by these TRGs. (B) The overall survival analysis of 7 TRGs in HNSCC was from the Kaplan–Meier plotter. (C) Tumor grade and N stage. (D) The forest plot of clinicopathologic characters processed by univariate Cox regression analysis and multivariate Cox regression analysis. HNSCC = head and neck squamous cell carcinoma, TRGs = T-cell proliferation-related genes.

Each HNSCC case’s survival status distribution and the risk scores are depicted in Fig. 3C and D; these data indicate that higher risk has worser outcome. High-risk group displayed significant decrease in survival than low-risk group (Fig. 3E, *P* < .001). We used chi-square tests on stage, grade, sex, age, and risk score for assessing whether the clinicopathological traits of HNSCC patients is correlated with the risk model. The heat map displays gene expression profiles (Fig. 4A). The prognostic associations of CYP27A1, EPO, FADD, IGF1, IL12RB2, TYK2, and ZAP70 were further determined according to the Kaplan–Meier Plotter analysis of overall survival (Fig. 4B). We found that patients with high expression of CYP27A1, EPO, IL12RB2, TYK2, and ZAP70 have better prognosis compared with low expression. However, worse prognosis was waiting for patients with high expression of FADD. Furthermore, N stage and tumor grade were very tightly related to risk (Fig. 4C). Univariate Cox regression analysis showed that risk score (HR = 1.769, 95% CI = 1.501–2.085, *P*-value < .001), age (HR = 1.026, 95% CI = 1.014–1.038, *P*-value < .001) were statistically significant, while the result of multivariate Cox regression analysis is that risk score (*P*-value < .001, 95% CI = 1.544–2.143, HR = 1.819), age (*P*-value < .001, 95% CI = 1.012–1.039, HR = 1.025), which demonstrate they were independent prognostic risk factors (Fig. 4D).

### 3.5. Correlation among the risk model and TICs

**Figure 5.**
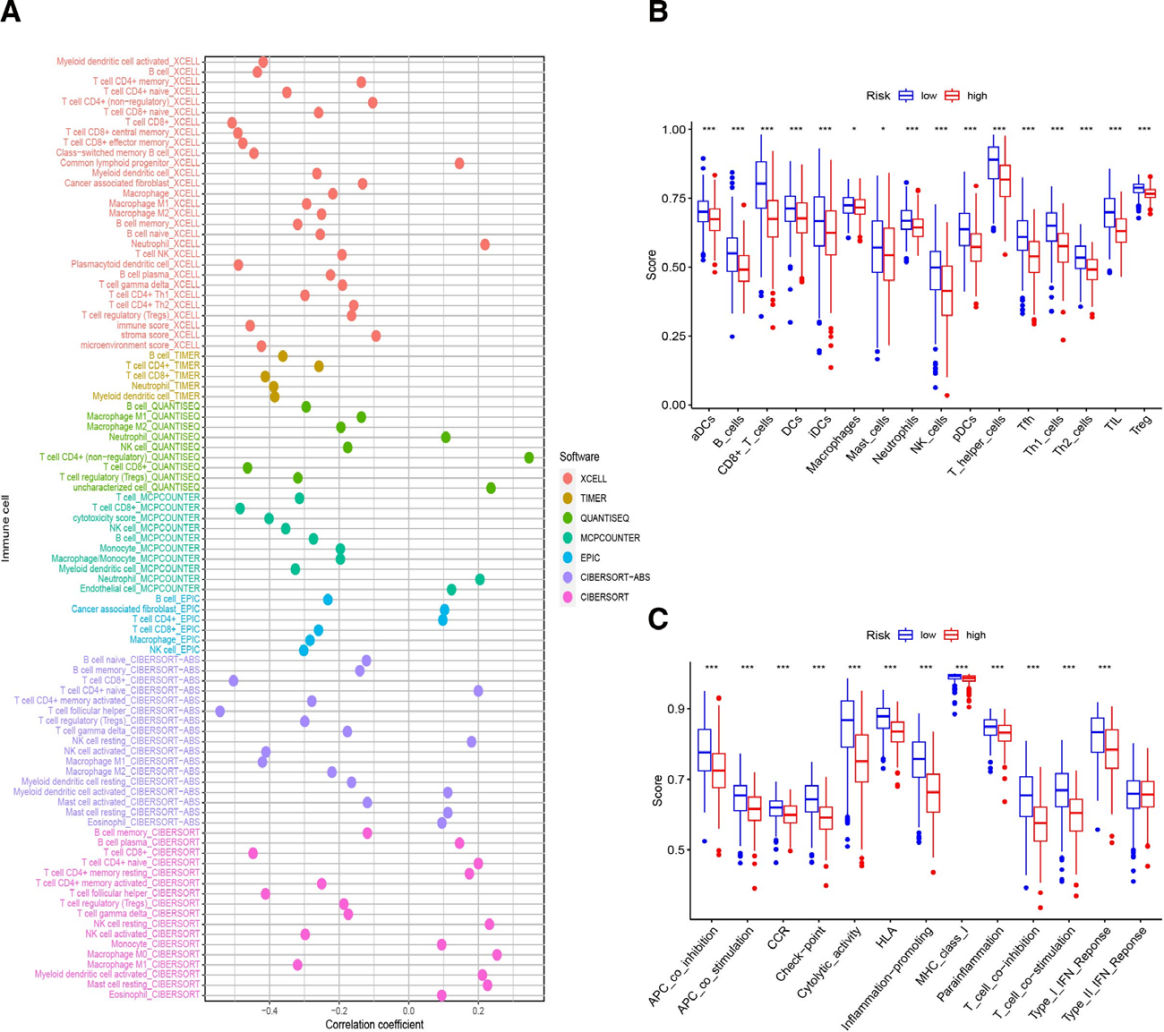
Evaluation of TICs by the risk model. (A) TICs such as macrophages, cancer-associated fibroblasts, neutrophils were more positively associated with patients in the high-risk group, as shown by Spearman correlation analysis. (B and C) Infiltrating scores of 16 immune cells and 13 immune-related pathways in various risk groups on the basis of TCGA cohort. TCGA = the cancer genome atlas, TICs = tumor-infiltrating immune cells.

**Figure 6.**
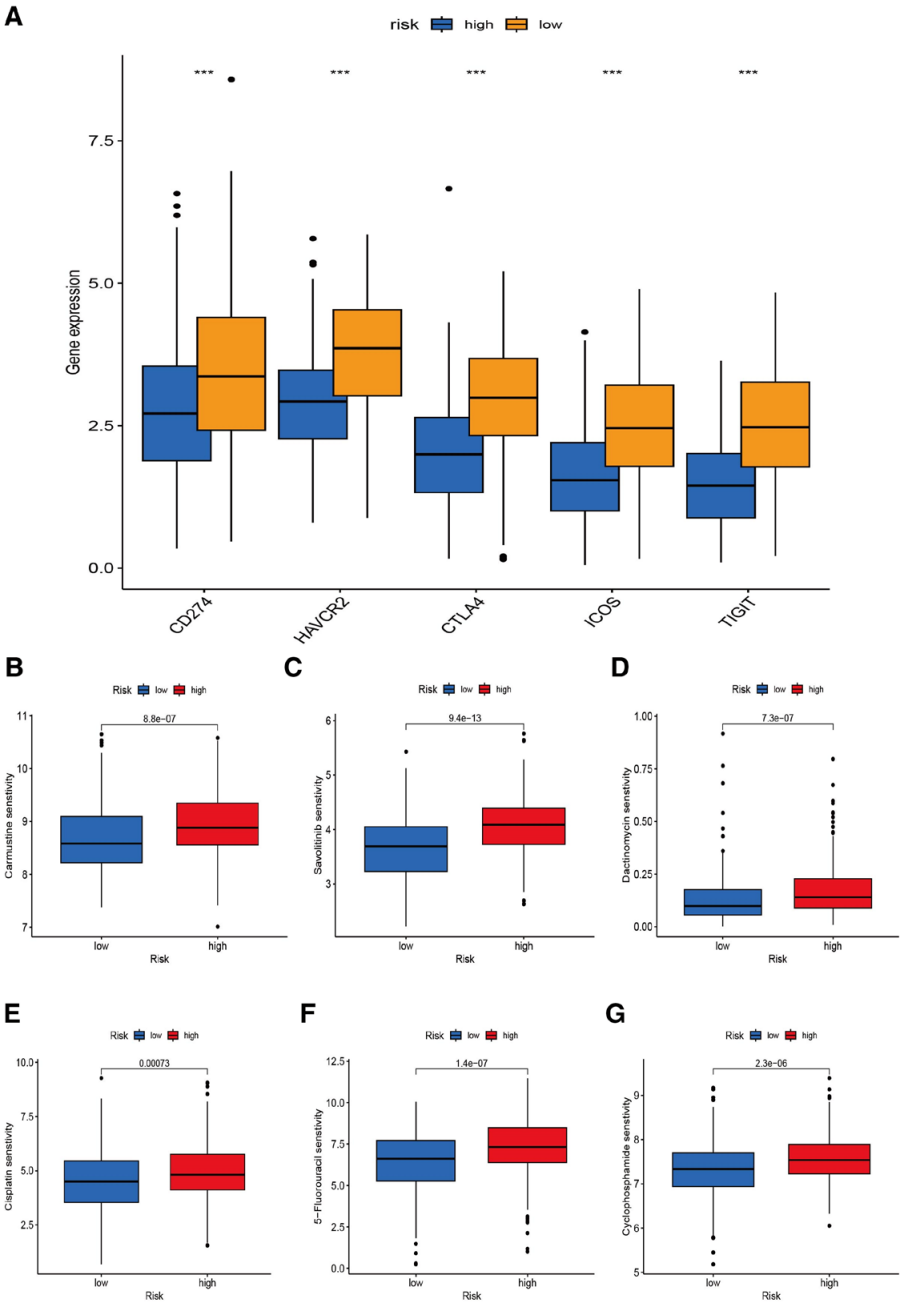
Immune check-point and chemotherapy drugs sensitivity investigation between risk groups. (A) The comparison of check-points expression between risk groups; (B–G) the comparison of several common chemotherapy drugs sensitivity between risk groups.

We concentrated on the model’s link with the tumor immunological microenvironment. We used Spearman correlation analysis and found that TICs were correlated positively with high-risk score, including cancer-related fibroblasts, macrophages, neutrophils. By contrast, B cells, CD8+, CD4 + T-cells and NK cells were correlated negatively (Fig. 5A). Using ssGSEA, we calculated the enrichment scores of distinct immune cell subsets, pathways and related function to investigate the relationship between risk scores and immunological state. Compared with the low-risk group, the accumulation of various immune cells (B cells, CD8 + T-cells, iDCs, Neutrophils, NK_cells, Tfh cells e.g.,) in the high-risk group are considerable different (Fig. 5B). Furthermore, immune function scores such as T-cell co-stimulation, Inflammation-promoting and Check-point in low-risk group were considerably higher than in high-risk group, suggesting that the immune function for tumor resistance was more active in low-risk group (Fig. 5C). In addition, we also assessed any possible correlation between the risk model and immune-associated biomarkers and observed a significant relation with the levels of CD274, HAVCR2, CTLA4, ICOS, and TIGIT (Fig. 6A).

### 3.6. Identification of 2 subtypes of T-cell proliferation-associated regulator

**Figure 7.**
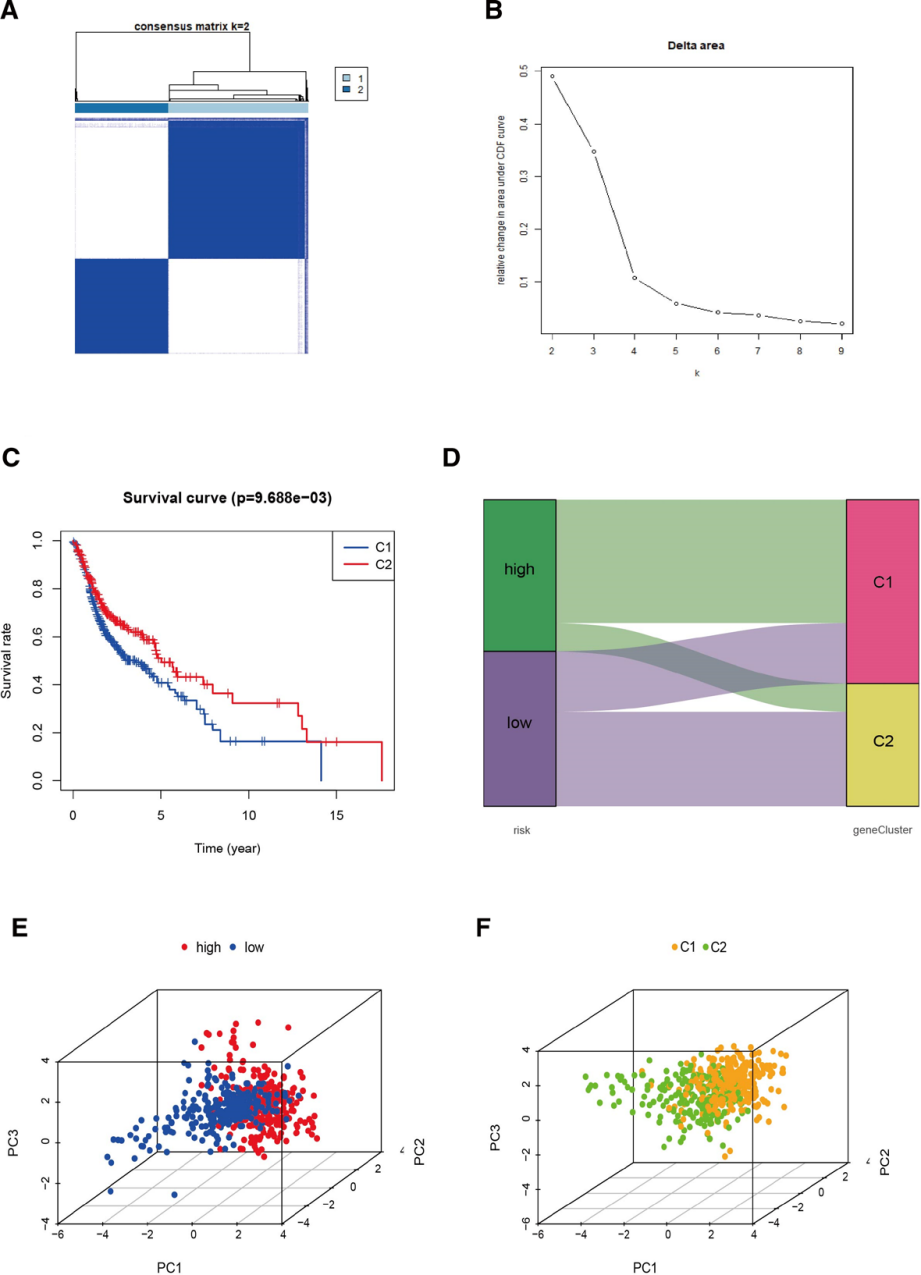
Distinction between risk groups and clusters. (A) Consensus clustering matrix for *k* = 2; (B) CDF plot; (C) overall survival curve of HNSCC patients in 2 clusters; (D) risk groups and clusters’ Sankey diagram (E and F) the 3D PCA of clusters and risk groups. CDF = cumulative distribution function, HNSCC = head and neck squamous cell carcinoma, PCA = principal component analysis.

**Figure 8.**
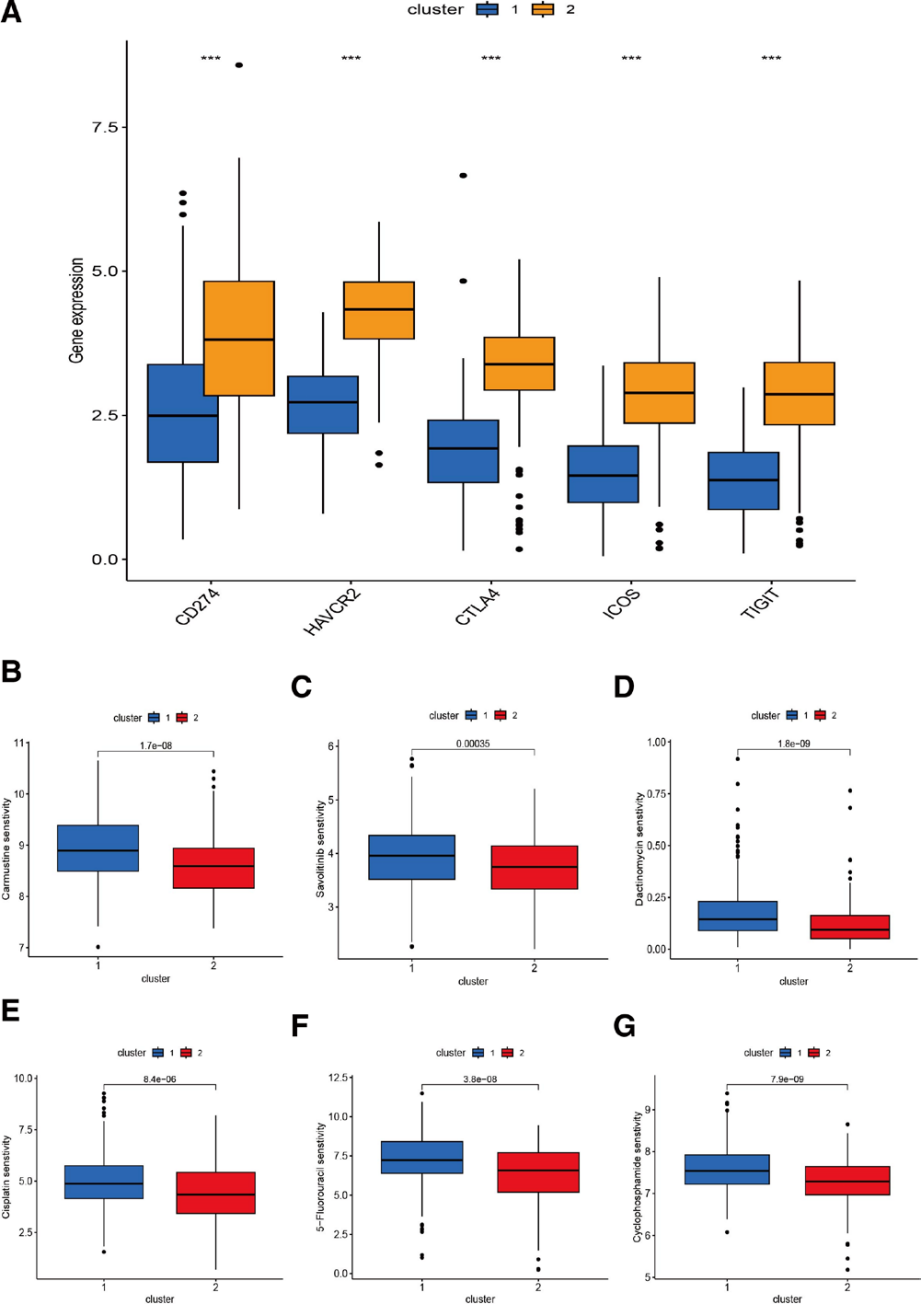
Immune checkpoint and chemotherapy drugs sensitivity assessment between clusters. (A) The comparison of checkpoints expression between clusters; (B–G) the comparison of several common chemotherapy drugs sensitivity between clusters.

We used consensus clustering (CC) analysis on 499 HNSCC suffers for investigating the connection among HNSCC subtype and the expression of 139 DETRGs. We changed the clustering variable (k) from 2 to 9 and found low inter-group correlations and the highest intragroup correlations when k = 2 (two subclasses were designated as cluster 1 and cluster 2), demonstrating that the 499 HNSCC suffers could be well devided into 2 clusters on the basis of TRGs (Fig. 7A). The maximum stability is indicated by the value of k determined by the cumulative distribution function plot, where the distribution approaches an approximate maximum (Fig. 7B). HNSCC patients belong to cluster 1 exhibited worse survival than cluster 2 (Fig. 7C). We perform the Sankey diagram and 3D PCA to contrast the differences and similarities among risk groups and clusters. High-risk group patients were mostly part of in Cluster1, while low-risk group patients mostly belong to the Cluster2 (Fig. 7D). 3-dimensional PCA could show the variances more intuitive between clusters as well as groups (Fig. 7E and F). Additionally, any possible correlation between the clusters and immune-associated checkpoints were observed and a significant relation with the levels of CD274, HAVCR2, CTLA4, ICOS and TIGIT were shown in Fig. 8A.

### 3.7. Analysis of the correlation between chemosensitivity and different groups

We investigated common medication sensitivities in individuals with low and high-risk ratings as well as cluster 1 and 2 to see if the efficacy of several regularly used chemotherapeutic medicines is associated with different risks and clusters. The patients with high-risk had a higher sensitivity for Carmustine (*P* = 8.8×10^−07^; Fig. 6B), Savolitinib (*P* = 9.4×10^−13^; Fig. 6C), Dactinomycin (*P* = 7.3×10^−07^; Fig. 6D), Cisplatin (*P* = .00073; Fig. 6E), 5-Fluorouracil (*P* = 1.4×10^−07^; Fig. 6F) and Cyclophosphamide (*P* = 2.3×10^−06^; Fig. 6G). In addition, the patients in cluster 1 also had a higher sensitivity for Carmustine (*P* = 1.7×10^−08^; Fig. 8B), Savolitinib (*P* = .00035; Fig. 8C), Dactinomycin (*P* = 1.8×10^−09^; Fig. 8D), Cisplatin (*P* = 8.4×10^−06^; Fig. 8E), 5-Fluorouracil (*P* = 3.2×10^−08^; Fig. 8F) and Cyclophosphamide (*P* = 7.9×10^−09^; Fig. 8G). Therefore, we could combine the risk with cluster to choose the more appropriate chemotherapeutic drugs for clinical treatment.

### 3.8. Gene mutation analysis

**Figure 9.**
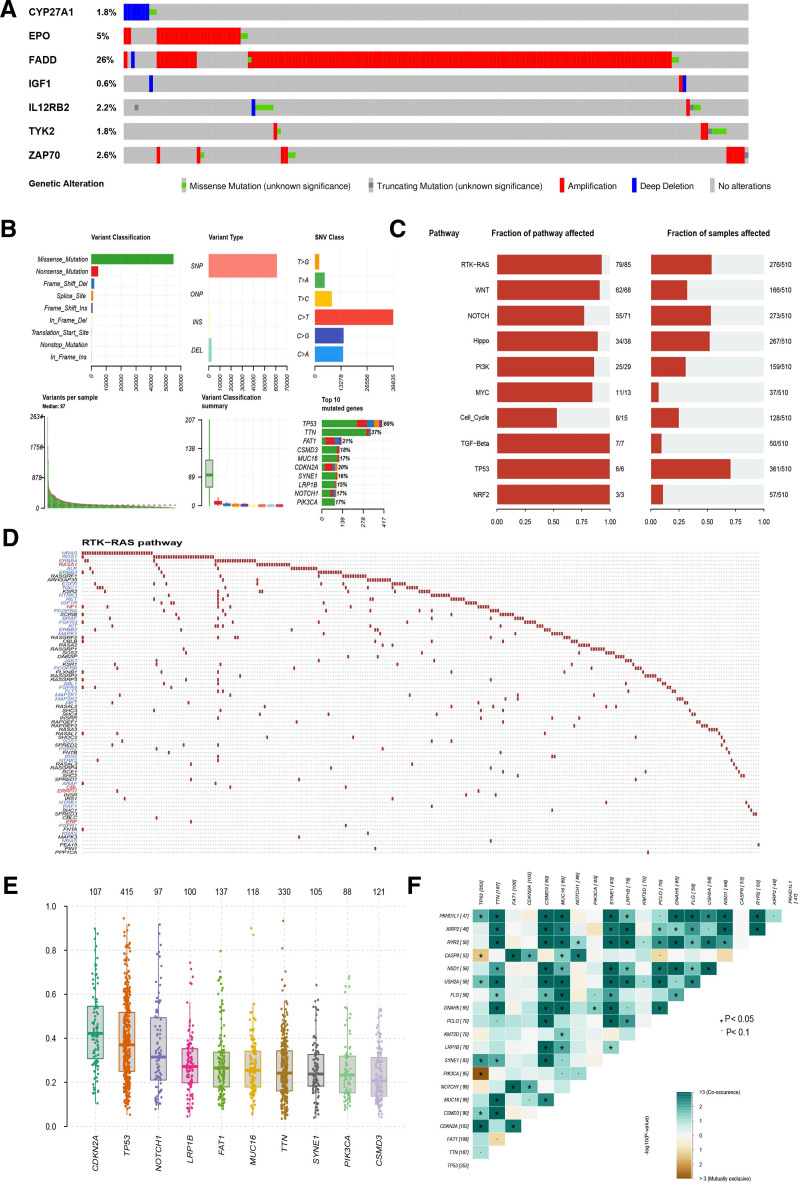
Gene mutation analysis of HNSCC. (A) The mutation of 7 TRGs acquired from cBioPortal database; (B) the mutation of HNSCC patients in TCGA database; (C) function enrichment of well-known Oncogenic Pathways based on TCGA cohorts; (D) visualization of RTK-RAS pathway, oncogenes are in blue and tumor suppressor genes are in red front; (E) the boxplot of VAF; (F) the relationship among genes mutually exclusive mutation or simultaneous mutation. HNSCC = head and neck squamous cell carcinoma, RTK-RAS = receptor tyrosine kinase-RAS, TCGA = the cancer genome atlas, TRGs = T-cell proliferation-related genes, VAF = variant allele frequencies.

cBioPortal database was used for analyzing mutations of the 7 TRGs involved in this model. FADD were significant mutation among these 7 genes (Fig. 9A). Furthermore, all somatic mutations information were analyzed and visualized. The highest mutation rate biomarker is TP53 and the most common variant classification is the missense mutation (Fig. 9B). The enrichment of known oncogenic signaling pathways in TCGA cohorts were shown in Fig. 9C and the receptor tyrosine kinase-RAS is the most significant enriched pathway. We visualized the receptor tyrosine kinase-RAS pathway, tumor suppressor genes are in red, and oncogenes are in blue font (Fig. 9D). Variant allele frequencies were mostly at low level (Fig. 9E). TP53 and CDKN2A are often co-mutated, while TP53 and PIK3CA are mutually exclusive mutants (Fig. 9F).

**Figure 10.**
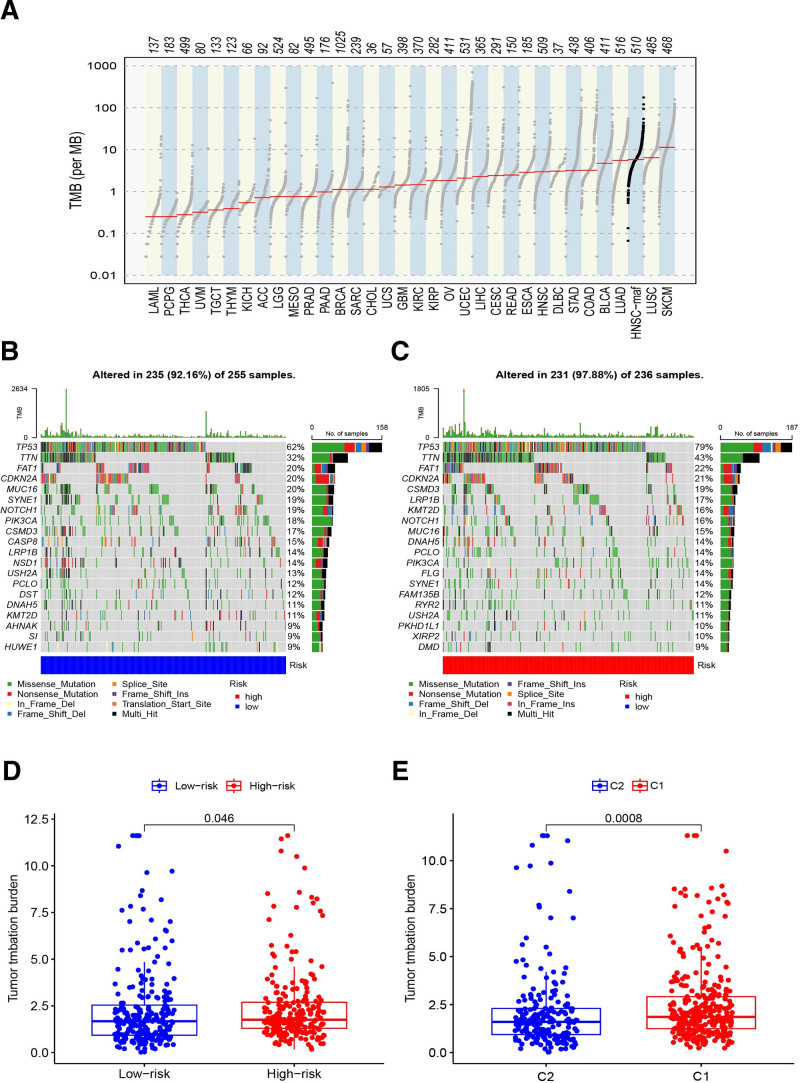
TMB of HNSCC patients. (A) The contrast of TMB between HNSCC and other types of tumors in TCGA database; (B) the TMB of high-risk group; (C) the TMB of low-risk group; (D) the contrast of TMB between different risk groups. (E) The contrast of TMB between different clusters. HNSCC = head and neck squamous cell carcinoma, TCGA = the cancer genome atlas, TMB = tumor mutation burden.

Tumor mutational burden is a known cause of carcinogenesis and progression and a biomarker for patients who benefit from immunotherapy and immune inhibition’s efficacy could be predicted by TMB. In contrast, the TMB of HNSCC was higher than the average level of other tumors (Fig. 10A). As demonstrated in Figure 10B and C, the top 5 mutation frequencies were TP53 (62%), TTN (32%), FAT1 (20%), CDKN2A (20%), and MUC16 (20%) in low-risk group. And TP53 (79%), TTN (43%), FAT1 (22%), CDKN2A (21%) and CSMD3 (19%) in high-risk group. TP53 is a well-studied oncogene, and the mutations of TP53 could participate in the pathological process and progression of multiple carcinomas.^[9]^ Therefore, we speculate that the high-risk patients’ immunosuppression might be associated with high mutations in TP53. We compared the results among 2 risk groups and between different clusters, deduced that TMB scores in Cluster 1 and high-risk groups were both higher (Fig. 10D and E).

## 4. Discussion

The mRNA levels of 139 putative TRGs in HNSCC and normal tissues were compared in our study. HNSCC could be divided into 2 subtypes based on these TRGs. Furthermore, we used univariate COX, multivariate COX and LASSO regression analysis to create risk profiles for 7 genes, which we then validated. Finally, we looked at survival, clinical features, gene mutation and immune cell infiltration in the novel model.

It is well known that T cells have pivotal influence in the incidence and progression of carcinoma. For HNSCC, immunotherapy and targeted therapy are implemented in immune cells. Due to the feature of regulating immune cell proliferation, the significance of TRGs is self-explanatory in antitumor. In our research, we developed a model in view of TRGs so far discovered. Our model concludes 7 TRGs (CYP27A1, EPO, FADD, IGF1, IL12RB2, TYK2, and ZAP70).

CYP27A1 was regarded as biomarker participates in cancer pathology in several literatures.^[10–12]^ This biomarker encodes a member of the cytochrome P450 superfamily of enzymes. The cytochrome P450 proteins are monooxygenases which catalyze many reactions involved in drug metabolism and synthesis of cholesterol, steroids and other lipids. Furthermore, CYP27A1 participate in synthesizing vitamin D.^[13]^ In addition, B cells, T cells, antigen-presenting-cells and other immune cells both present vitamin D receptors.^[14]^Therefore, CYP27A1 may influence the synthesis of vitamin D and further regulate the T cells to affect the tumor immune microenvironment of HNSCC.

EPO had been reported as a hypoxia-related and immune-associated biomarker in many cancers.^[15,16]^ This protein is mainly synthesized in the kidney, secreted into the blood plasma, and binds to the erythropoietin receptor to promote red blood cell production, or erythropoiesis, in the bone marrow. Expression of this gene is upregulated under hypoxic conditions. In addition, previous literature demonstrated that EPO could regulate immune cells, such as promoting Treg cells proliferation.^[17]^ Therefore, EPO is not only the signature of hypoxic tumor microenvironment but also the biomarker of T-cell proliferation.

FADD encode gene which interacts with various cell surface receptors and mediates cell apoptotic signals. Through its C-terminal death domain, this protein can be recruited by TNFRSF6/Fas-receptor, tumor necrosis factor receptor, TNFRSF25, and TNFSF10/TRAIL-receptor, and thus it participates in the death signaling initiated by these receptors. The pathogenesis of numerous types of cancer have been demonstrated strongly correlated with FADD dysregulation.^[18]^ However, the dominant function of FADD is inhibition of programmed necrosis during T-cell proliferation.^[19]^

IGF1 is a member of a family of proteins involved in mediating growth and development.^[20]^ High expression of IGF1 in HNSCC have been demonstrated by qRT-PCR and western-blot than normal tissue,^[21]^ which is consistent with Figure 5A , IGF1 is highly expressed in high-risk group. Antiapoptotic is the most obvious feature of tumor cell and IGF1 have been validated the strong activity of antiapoptotic in several researches.^[22]^Due to IGF1’s stimulating proliferation and antiapoptotic, T-cell and tumor cell are all benefit from IGF1.

Tyrosine kinase 2 (TYK2) is a member of the Janus kinase (JAK) family and is involved in immune and inflammatory signaling. Our research group have found that knockdown of TYK2 could significantly promote the proliferation, migration, and invasion of HNSCC cell lines in vitro and have published the results.^[23]^

When T-cell executive functions, T-cell antigen receptor (TCR) is required. ZAP70 is required to participate in signal transduction from the TCR as cytoplasmic tyrosine kinases.^[24]^ Furthermore, ZAP70 was reported as immune-related prognostic biomarkers in laryngeal cancer, which is the 1 type of the HNSCC.^[25]^

The IL-12 receptor is composed of the β1 and β2 chains, both of which are needed for high-affinity binding of the cytokine and initiation of signal transduction.^[26]^ The low expression of IL-12RB2 induces to autoimmune disease and cancer.^[27]^This is match to Figure 5A, IL12RB2 is low expression in high-risk group. Additionally, IL12RB2 was demonstrated that it creates a homeostasis within the tumor cells and tumor-infiltrating lymphocytes and this homeostasis affects prognosis in laryngeal cancer.^[28]^ Pleiotropic cytokine Interleukin 12 (IL-12) has a significant influence in against cancer through Th1 immune response.^[29]^ Therefore, we deduced that IL12RB2 may mediate IL12 to influence Th cells to participate in the cancer resistance.

Tumor immunotherapy was regarded as a breakthrough in cancer treatment. On the other hand, immunotherapy response differs between malignancies and within cancer Cohorts.^[30]^ As a result, TMB levels were established to predict cancer patients’ response to immunotherapy, and high TMB has been demonstrated to correlate with immunotherapy efficacy.^[31]^ CD4+, CD8 + T-cells and other antitumor immune cells were in negatively correlation with score, while cancer-associated fibroblasts (CAF) and the score were positive correlation, which may have interpreted the poor prognosis of HNSCC suffers. A longer overall survival in HNSCC may be related with the CD4 + T-cells’ high infiltration. Patient responses and multiple preclinical cancer models have demonstrated that healing effect of check-point blocking immunotherapy is closely related with the status and quantity of CD8 + T-cells.^[32]^ Several literatures have demonstrated that CAF promote tumor growth, invasion and metastasis.^[33]^ Immunotherapy-related biomarker analysis showed that the expression levels of immune check-point molecules such as CD274, HAVCR2, CTLA4, ICOS, and TIGIT were significantly upregulated in the low-risk group and cluster 2 compared with the high-risk group and cluster 1. In addition, inter-cluster variation in expression levels of immune check-point is more pronounced than inter-risk group variation, suggesting that patients in the cluster 2 group may be more effective for immune check-point inhibitors therapy. Finally, the immunoassay results could help clinicians personalize immunotherapy by elucidating the immune mechanisms by which T cell proliferation -related signatures affect HNSCC patients’ prognosis.

In addition to immunotherapy, we explored the efficacy of some common chemotherapeutic agents in different risk groups and clusters. We found the *P*-value of different sensitivity in clusters is smaller than in risk groups except Savolitinib. We found not only the check-point inhibitors but also the chemotherapeutic drugs both have more obvious differences between inter-cluster variation than differences between inter-risk groups. Therefore, in the 7 TRG-based clusters, precision drug therapy and immunotherapy are most possible to improve patient prognosis and outcomes.

Our models based on the TRGs in HNSCC could quantify a patient’s risk of HNSCC by combining multiple genetic loci to identify high-risk individuals for early intervention and help enable precision medicine, selecting drugs or adjusting doses based on genotype (pharmacogenomics). The most significant implication is to improve identifying the risk of patients with HNSCC accuracy. While traditional single-gene testing may be limited in HNSCC, polygenic modeling can improve accuracy and specificity and reduce miscarriage by combining genetic information and other biomarkers.

However, we also realize several limitations of this study. First, our current results are only available on public databases. More experiments required to construct prognostic models, such as molecular mechanisms of T cell proliferation -related biomarkers, need to be further validated in experimental studies. Second, because the dataset for the analysis was required by TCGA, it was insufficient. While we employed several approaches to confirm the validity and accuracy of this predictive model, more external datasets will be required to confirm it. Finally, the risk scores in predicting HNSCC prognosis and immunotherapy should also be confirmed. Therefore, in our future work, we will analyze the postoperative pathological tissues of HNSCC patients, typify patients according to the differences in the expression of the 7 TRGs in the pathological tissues, and design individualized immunotherapy for different risks of HNSCC patients to ensure the quality of their prognosis.

## 5. Conclusions

In conclusion, our research identifies a unique prognostic TRG signature effective in customized risk classification and survival prediction in HNSCC patients. The signature has a good prediction power for diverse clinical subgroups and is closely related to clinicopathological characteristics. The TRG prognostic profile, in particular, is substantially linked with immune-related processes, demonstrating that T cell proliferation has irreplaceable status in immunotherapy. Understanding the underlying mechanisms of T cell proliferation as well as its impact on survival, and its implications for HNSCC treatment may provide insights into identifying HNSCC therapeutic targets.

## Author contributions

**Conceptualization:** Wenkai Huang.

**Data curation:** Wenkai Huang.

**Funding acquisition:** Yuanyin Wang.

**Methodology:** Wenkai Huang.

**Project administration:** Lin Yang, Ran Chen.

**Software:** Mingyu Zhao.

**Validation:** Yunshan Li.

**Visualization:** Junwei Xiang.

**Writing – original draft:** Wenkai Huang.

## Supplementary Material


